# Special Issue “Antifungal Drug Discovery: Progresses, Challenges, Opportunities”

**DOI:** 10.3390/ijms26052065

**Published:** 2025-02-27

**Authors:** Bernhard Biersack

**Affiliations:** Organic Chemistry Laboratory, University of Bayreuth, Universitätsstrasse 30, 95440 Bayreuth, Germany; bernhard.biersack@yahoo.com

The considerable health risks associated with fungal infections are continuously rising, thereby requiring proper and efficient antifungal treatment options [1]. However, the current clinical arsenal of antifungal drugs for the therapy of human mycoses is limited and comprises only a few compound classes, e.g., azoles, echinocandins, polyenes, and the antimetabolite flucytosine. Inactivity, resistance formation, and toxic side effects are the major drawbacks of the currently approved antimycotics; thus, the development of new antifungal drugs is mandatory [2].

Human pathogenic fungi and the affected patient groups are highly diverse, which requires tailor-made treatments. Immunocompromised people (e.g., elderly people, organ transplantation and cancer patients, and persons infected with HIV or tuberculosis) especially suffer from life-threatening systemic fungal infections such as candidiasis, cryptococcal meningitis, and aspergillosis [3]. The formation of drug-resistant pathogenic fungal strains, as well as the emergence of new highly problematic species such as *Candida auris*, poses a considerable global health threat, warranting the development of new and efficient antifungal drug candidates with limited side effects [4,5]. The severe skin mycoses eumycetoma, chromoblastomycosis, and sporotrichosis are classified as neglected tropical diseases (NTDs), which also appear in younger people [6]. Potent antifungals still need to be discovered for these fungal NTDs because the existing drugs are often inefficient or unavailable for patients in remote rural tropical and subtropical regions [7].

The Special Issue “Antifungal Drug Discovery: Progresses, Challenges, Opportunities” was launched in August 2023 and covers the latest developments and state of the art in antifungal drug discovery. Several eminent experts in the field have disclosed innovative therapies in this Special Issue. Six research and three review articles have been published, and their contents are briefly summarized herein. For more details, I strongly recommend reading the original full-length open access articles.

Prominent ingredients of the tea plant *Camellia sinensis* such as the flavonoid epigallocatechin-3-gallate (EGCG) are known for their antifungal activities [8]. Triterpene saponins isolated from *Camellia* species have also exhibited considerable biological activities [9]. The tea seed triterpene saponins assamsaponin A and theasaponin E1 have shown activity against *C. albicans* (including fluconazole-resistant strains) by disrupting cell membrane integrity and interfering with lipid and energy metabolism [10]. Chen et al. investigated the effects of assamsaponin A, theasaponin E1, and theasaponin E2 on *C. albicans* biofilm formation [11]. Notably, assamsaponin A and theasaponin inhibited *C. albicans* adhesion on polystyrene surfaces at low concentrations (1/8 of their minimum inhibitory concentration/MIC). Mechanistically, assamsaponin A and theasaponin E1 suppressed various virulence factors as a consequence of cAMP-PKA and MAPK signaling downregulation by RAS1 inhibition, while theasaponin E2, which displayed no antifungal activity, reduced *C. albicans* adhesion and regulated fungal morphology and hyphal growth in a RAS1-independent way. The inhibitory effects of theasaponins on *C. albicans* growth, viability, and biofilm formation were enhanced upon their combination with the natural antioxidant ascorbic acid (also known as vitamin C) via redox imbalance and energy deficiency mechanisms [12]. Thus, tea seed theasaponins can become valuable medical tools for the treatment of candidiasis. The growing importance of triterpenoid saponins from tea plants as antimycotic agents was recently supported by the isolation of new saponins from tea flowers, which showed anticandidal activity either equal or superior to fluconazole [13].

Microbes growing under extreme conditions have become an important source of bioactive natural products [14]. Giordano et al. studied the antifungal activity of the crude ethyl acetate extract and its active ingredients obtained from the Antarctic *Streptomyces albidoflavus* CBMAI 1855 bacterium [15]. The crude extract exhibited broad antifungal activity against various *Candida*, *Aspergillus*, and *Cryptococcus* species, including drug-resistant clinical isolates (MIC and MFC values between 1.5 and 3 mg/mL). Co-cultivation with *Aspergillus flavus* was applied to identify the active and structurally diverse antifungal metabolites of *S. albidoflavus* [e.g., antimycin A, fungimycin, surugamides, 9-(4-aminophenyl)-3,7-dihydroxy-2,4,6-trimethyl-9-oxo-nonoic acid, and ikarugamycin], and inhibition zones were used for metabolomic analysis. It was found that co-cultures with the pathogenic *A. flavus* fungus induced the biosynthesis of some antifungal metabolites in *S. albidoflavus*, which could not be isolated from pure *S. albidoflavus* cultures. Certain biosynthetic gene clusters were identified, which should be explored in more detail in the future to isolate new potent antifungals from this Antarctic bacterium.

The screening of established and available compound libraries for antifungal activity is a promising strategy for developing antimycotic drug candidates, in particular; this is important for the therapy of fungal NTDs such as eumycetoma, where the costly development of new drugs is very limited. In 2019, Medicines for Malaria Venture (MMV) and Drugs for Neglected Diseases initiative (DNDi) launched the open access Pandemic Response Box drug library, which was meanwhile also tested for anti-mycetoma activity, leading to the identification of olorofim, ravuconazole, and benzimidazole carbamates as promising drugs with in vivo activity [16]. Since then, Ma et al. screened the MMV Global Health Priority Box for activity against *Madurella mycetomatis* and *Falciformispora senegalensis*, the main causative agents of eumycetoma [17]. The pyrazolopyrimidine derivative MMV1804559 was active in vitro against both aforementioned fungi and was consequently selected for in vivo testing using a *Galleria mellonella* model infected with *M. mycetomatis*. Notably, MMV1804559 prolonged the survival of infected *G. mellonella* larvae and suppressed the formation of the characteristic black grains built by the fungus at advanced stages of infection. These are very promising results for this heterocyclic compound from an MMV library, and further studies will determine whether MMV1804559 can become a new drug for eumycetoma therapy.

Antifungal peptides are short cationic peptides with significant levels of activity against various pathogenic fungi [18]. The palindromic RWQWRWQWR (R-1-R) oligopeptide was derived from bovine lactoferricin and exhibited antifungal activity against sensitive and resistant *C. albicans* and *C. auris* in combination with *Bidens pilosa* ethanol extract [19]. Inspired by the remarkable antifungal effects of R-1-R, Vargas-Casanova et al. have disclosed new insights into the complex mechanisms of action of R-1-R alone or in combination with *B. pilosa* extract in sensitive and fluconazole-resistant *C. albicans* models using proteomics [20]. A predominant downregulation of protein expression was observed upon combination therapy in contrast to treatment with R-1-R alone, which underlines the beneficial role of *B. pilosa* extract in the sensitization of fungal cells to R-1-R. The combination of R-1-R with *B. pilosa* extract downregulated proteins associated with cell wall biology (cytoskeleton proteins, a chitinase, a cell wall integrity protein, a protein involved in ergosterol synthesis, and a sterol transfer protein), membrane transport (carbohydrate transport and MDR1 transporters), oxidative stress (e.g., oxidoreductases), mitochondria, and nucleic acid biology (ribosome function, rRNA maturation, and nucleocytoplasmic transport).

The scorpion venom-derived peptide ToAP2 was shown to kill *C. albicans* planktonic cells based on severe changes in the fungal cell wall morphology, including cell wall deformations [21]. A follow-up research article to this finding by do Nascimento Dias et al. deals with the ToAP2-mediated antibiofilm activity in more detail [22]. The combination of ToAP2 with fluconazole led to synergy effects on early and mature phases of biofilm formation and the reduced viability of fungal cells within the treated biofilms. Scanning electron microscopy and atomic force microscopy analyses revealed damaged *C. albicans* biofilm structures, including cell collapse and membrane roughening. ToAP2 plus fluconazole also suppressed *C. albicans* biofilms on the surface of infusion tubes and polyurethane catheters. In addition, the drug combination altered the expression of virulence factors. For instance, the expression of ERG11 involved in ergosterol biosynthesis was downregulated.

In addition to the described original research, three review articles were published in this Special Issue. Herbal antifungals are promising and cost-effective drug candidates for the therapy of various mycoses [23]. Picheta et al. have summarized the state of the art of current phytotherapy for the management of vulvovaginal candidiasis (VVC), which is a common health problem for pre-menopausal women [24]. Extracts from *Allium jesdicarium* and *Fridericia chica*, as well as the seed oil of dill (*Anethum graveolens*), were reported to show significant antifungal activities. The pronounced antimycotic properties of the alkaloid berberine (from *Berberis vulgaris*), the phenolic derivative curcumin (from turmeric, *Curcuma longa*), the organosulfur compound allicin (from *Allium sativum*), and the cannabinoid cannabidiol (from *Cannabis sativa*) were also outlined in this review. All in all, this review provides several clues in favor of phytotherapy as a suitable treatment option for VVC.

The natural product curcumin, which is found in considerable amounts in turmeric, exhibited a plethora of biological activities [25]. Since curcumin absorbs blue light, it can be applied for antimicrobial photodynamic therapy (aPDT); Kubizna et al. have systematically reviewed the effects of curcumin-based aPDT on oral candidiasis [26]. The great number of in vivo studies showcasing the promising aPDT potential of curcumin in laboratory animals is of particular interest. In addition, a clinical study using curcumin-mediated aPDT in patients suffering from stomatitis revealed promising clinical data on the efficacy of this therapy option in humans, which should be confirmed in future clinical trials. The characteristics of curcumin samples and light sources for curcumin-based aPDT were also discussed in this review. Thus, curcumin plus light irradiation can become a proper therapy for oral candidiasis in the future.

The synthetic salicylanilide niclosamide has been applied for decades for the therapy of worm infections in adults and children [27]. However, niclosamide was meanwhile also repurposed for the treatment of other infectious diseases including microbial and viral diseases [28]. My contribution to this Special Issue covers the multiple and considerable antifungal activities of niclosamide and other structurally related salicylanilides [29]. For instance, niclosamide was active against *C. albicans* (including azole-resistant strains), *Cryptococcus neoformans*, *Trichophyton tonsurans*, and fungi causing severe skin NTDs such as mycetoma (*M. mycetomatis*) and sporotrichosis (*Sporothrix brasiliensis*) based on mechanisms that ultimately inhibit fungal growth, survival, and biofilm formation. Various anthelmintic salicylanilides currently applied in veterinary medicine, including oxyclozanide, 3,5-diiodosalicylanilides (rafoxanide and closantel), and bromsalans, also displayed antifungal activities against various human pathogenic fungi (*Aspergillus* sp., *C. albicans*, and *T. tonsurans*) and have the potential to become drugs for human mycoses. Moreover, several antifungal experimental salicylanilides were covered in this review, and structure–activity relationships were discussed.

All in all, this Special Issue covers a well-balanced collection of state-of-the-art topics in the prospering and competitive field of antifungal drug development, which can address and inspire clinicians, microbiologists, and medicinal chemists specifically among the readership of this journal (Figure 1).

## Figures and Tables

**Figure 1:**
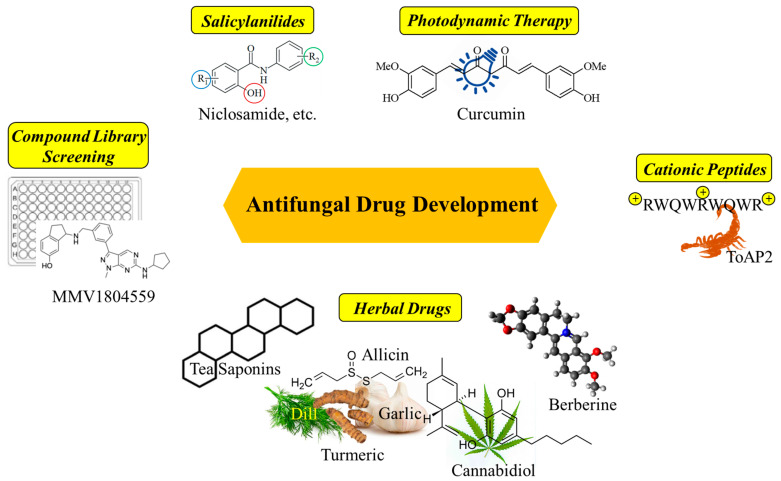
Topics covered in the Special Issue “Antifungal Drug Discovery: Progresses, Challenges, Opportunities” include various herbal drugs [11,12,24], cationic peptides [20,22], photodynamic therapy (with curcumin) [26], compound library screening [17], and salicylanilides [29].
